# Neurofilament Light Chain as a Biomarker of Global Cognition in Individuals With Possible Vascular Mild Cognitive Impairment

**DOI:** 10.1177/08919887241254469

**Published:** 2024-05-17

**Authors:** Amish Gaur, Damien Gallagher, Nathan Herrmann, Jinghan Jenny Chen, Susan Marzolini, Paul Oh, Yutaka Amemiya, Arun Seth, Alex Kiss, Krista L. Lanctôt

**Affiliations:** 1 Hurvitz Brain Sciences Program, 282299 Sunnybrook Research Institute, Toronto, ON, Canada; 2 Department of Pharmacology & Toxicology, 7938 University of Toronto, Toronto, ON, Canada; 3 Department of Psychiatry, 12366 University of Toronto, Toronto, ON, Canada; 4 KITE Research Institute, 7961 Toronto Rehabilitation Institute, Toronto, ON, Canada; 5 Genomics Core Facility, 282299 Sunnybrook Research Institute, Toronto, ON, Canada; 6 Department of Laboratory Medicine and Pathobiology, 7938 University of Toronto, Toronto, ON, Canada

**Keywords:** Alzheimer’s disease, mild cognitive impairment, vascular cognitive impairment, cardiac rehabilitation, neurofilament light chain, global cognition

## Abstract

**Background:**

Neurofilament Light Chain (NfL) is a biomarker of axonal injury elevated in mild cognitive impairment (MCI) and Alzheimer’s disease dementia. Blood NfL also inversely correlates with cognitive performance in those conditions. However, few studies have assessed NfL as a biomarker of global cognition in individuals demonstrating mild cognitive deficits who are at risk for vascular-related cognitive decline.

**Objective:**

To assess the relationship between blood NfL and global cognition in individuals with possible vascular MCI (vMCI) throughout cardiac rehabilitation (CR). Additionally, NfL levels were compared to age/sex-matched cognitively unimpaired (CU) controls.

**Method:**

Participants with coronary artery disease (vMCI or CU) were recruited at entry to a 24-week CR program. Global cognition was measured using the Montreal Cognitive Assessment (MoCA) and plasma NfL level (pg/ml) was quantified using a highly sensitive enzyme-linked immunosorbent assay.

**Results:**

Higher plasma NfL was correlated with worse MoCA scores at baseline (*β* = −.352, *P* = .029) in 43 individuals with vMCI after adjusting for age, sex, and education. An increase in NfL was associated with worse global cognition (*b*[SE] = −4.81[2.06], *P* = .023) over time, however baseline NfL did not predict a decline in global cognition. NfL levels did not differ between the vMCI (n = 39) and CU (n = 39) groups (*F*(1, 76) = 1.37, *P* = .245).

**Conclusion:**

Plasma NfL correlates with global cognition at baseline in individuals with vMCI, and is associated with decline in global cognition during CR. Our findings increase understanding of NfL and neurobiological mechanisms associated with cognitive decline in vMCI.

## Introduction

The global prevalence of dementia is estimated to increase considerably, with a projection of over 150 million cases by 2050.^1^ An increase in dementia prevalence of this magnitude threatens to overwhelm global health care systems. To prevent this increase, improved understanding of the underlying pathophysiology and trajectory of cognitive decline is critical. Alzheimer’s disease (AD) is generally considered to be the most prevalent cause of dementia, with vascular dementia (VaD) being the second most common underlying etiology.^2-4^ Several studies have focused on better understanding the early stages of AD, such as mild cognitive impairment (MCI) and preclinical AD.^5-10^ Yet, there is increasing evidence of high co-morbidity between AD and cerebrovascular disease,^11-16^ which highlights the importance of investigations focused on the neurobiological underpinnings of vascular cognitive impairment.

Post-mortem studies have reported that up to 50% or more of AD cases have underlying cerebrovascular pathology (eg, cerebral amyloid angiopathy, microinfarcts, small vessel disease), indicative of a high prevalence of dementia of mixed etiology.^11,15^ There is also an abundance of epidemiological evidence demonstrating that cerebrovascular risk factors, such as coronary artery disease (CAD), obesity, diabetes, hypertension, and hyperlipidemia can increase the risk of developing dementia, including AD.^16-22^ The neurobiological basis of this relationship continues to be elucidated but it is believed that vascular risk factors contribute to dysfunction of the neurovascular unit (NVU).^12,16,20,23^ The NVU is a collection of neurons, glial cells, and vascular cells, including those that make up the blood brain barrier. Alterations to the NVU can result in reduced cerebral blood flow triggering a cascade of events, including increased inflammation, oxidative stress, decreased amyloid-beta (Aβ) clearance with aggregation, and ultimately neurodegeneration.^12,16,23^ This sequence of events indicates that vascular disease can be an independent contributor to both AD pathogenesis and disease-associated neurodegeneration. However, further research in this area is needed, and may be greatly facilitated using novel accessible biomarkers.

While biomarker research in dementia has typically focused on Aβ and phosphorylated tau, the more recently developed neurodegeneration (‘N’) biomarker category (from the Aβ, neurofibrillary tangle, neurodegeneration or A/T/N biomarker framework described by Jack et al., 2016) has gained increasing interest.^24^ This category can increase insight into cognitive decline that is independent of Aβ and neurofibrillary tangles.^8,24^ Cerebrospinal fluid (CSF) total-tau, 18-F-fluorodeoxyglucose positron emission tomography, and structural magnetic resonance imaging are often used to classify the ‘N’ category,^24^ but there are also novel candidate biomarkers that can allow for a similar characterization of a neurodegenerative profile.^25^ For example, neurofilament light chain (NfL) is a protein subunit that is highly concentrated in neuronal axons and is released in high volumes in response to neuroaxonal injury.^26-28^ NfL can then be subsequently detected in body fluid compartments due to its high solubility. Consequently, NfL levels are found to be considerably elevated compared to health controls in several different neurodegenerative conditions (eg, AD and VaD).^27-30^ NfL levels in CSF and blood compartments are also highly correlated,^31-33^ demonstrating its potential to be a relatively easily accessible blood-based biomarker.

Many studies have investigated NfL in AD, but few have evaluated the clinical utility of blood NfL in the early stages of vascular cognitive impairment (VCI) using sensitive, easy-to-administer cognitive tests. Such studies may improve our neurobiological understanding of changes in cognition within a population of individuals that are vulnerable to both increased neurodegeneration and subsequent cognitive decline. In the present study, we therefore investigated the utility of blood NfL as a biomarker of global cognitive performance in individuals with vascular MCI (vMCI) in both cross-sectional and longitudinal analyses.

## Materials and Methods

### Study Design

The present study explored the relationship between blood NfL levels and cognitive performance in those with possible vMCI using both cross-sectional and longitudinal study designs. Blood NfL levels were also compared between participants with vMCI and an age and sex-matched cognitively unimpaired (CU) control group. All participants were recruited at the start of a 24-week cardiac rehabilitation (CR) program at the University Health Network Toronto Rehabilitation Institute, which consists of exercise and lifestyle education, aerobic exercise, and resistance training supervised by a health care professional.^34,35^ Specifically, the program involves a weekly group education session and a supervised exercise class in which patients perform moderate-intensity aerobic exercise (ie, walk or walk/jog), and resistance training using hand-held weights. Participants were prescribed personalized exercise prescriptions in which they were advised to complete aerobic exercise 4 additional times, including 1 to 2 resistance sessions at their home. Directly measured cardiorespiratory fitness (CRF; VO_2peak,_ mL⋅kg^−1^⋅min^−1^), was ascertained by symptom-limited graded exercise test at baseline and 24-weeks.^36,37^ Participant blood samples, demographic, and cognitive assessment data obtained at baseline (cross-sectional) and endpoint (longitudinal) were used as appropriate for all subsequent analyses. Written informed consent was obtained from participants prior to study enrolment. All study procedures were approved by the Research Ethics Boards of Sunnybrook Health Sciences Centre and the University Health Network.

### Study Participants

The present study enrolled individuals between 50-75 years old who were able to read and communicate fluently in English. Participants in the study had stable CAD, which was defined by 1 or more of the following: a history of myocardial infarction, coronary angiographic evidence of greater than 50% stenosis in at least 1 major coronary artery, or a prior revascularization procedure. All participants included in the study also had documented dyslipidemia and were consequently receiving statin therapy. Patients were excluded from the study if they were prescribed medications that may influence cognitive performance, such as hypnotics, antipsychotics, antidepressants, and anticholinergic agents. Furthermore, participants were excluded if they demonstrated severely disrupted liver, renal, and/or lung function, substance abuse, uncontrolled hypothyroidism, sepsis, autoimmune disorders, bipolar disorder, schizophrenia, or a previously diagnosed neurodegenerative illness including dementia of any subtype. In addition to a clinical diagnosis, the Mini-Mental Status Examination (MMSE) was used to screen for dementia, such that individuals with an MMSE score of <24 were excluded from the study.

Demographic information and clinical characteristics were obtained from study participants via patient interviews and/or health records that were available at study baseline. This information included age, sex, education, smoking history, body mass index (BMI; calculated as mass (kg)/height (m^2^)), psychiatric and neurological history, clinical co-morbidities, and the use of concomitant medications.

Participants were classified as possible vMCI based on criteria derived from Gorelick et al., 2011.^12^ The National Institute of Neurological Disorders and Stroke and Canadian Stroke Network 30-min standardized battery was used to evaluate cognitive performance.^38^ Cognitive impairment was defined as at least 1 standard deviation (SD) below population (eg, age and education-matched) norms on tests within a cognitive domain. Briefly, 4 cognitive domains were assessed, including verbal memory (California Verbal Learning Test-II), executive function (trail-making test B, controlled oral word association test), language (animal naming test), and visuospatial function (Brief Visuospatial Memory Test – Revised).^38-44^ A detailed explanation of each cognitive test used can be found in the Supplemental Material provided. Information was insufficient for the diagnosis of ‘probable’ vMCI due to absence of neuroimaging in this study, and so participants were classified as ‘possible’ vMCI based on their clinical profile (ie, cognitive impairment and significant co-morbid vascular risk factors) supporting an increased likelihood of impairment due to underlying vascular disease.^12,23,45^ In addition, a CU control group was included in the cross-sectional analysis, which consisted of CR participants who performed normally on neuropsychological testing, had a similar clinical vascular profile, and were age (±5 years) and sex-matched with the vMCI group.

### Global Cognition

Global cognition was the primary cognitive outcome of this study and was assessed using the Montreal Cognitive Assessment (MoCA).^46^ The MoCA is a brief (10-minute), easy-to-administer cognitive assessment that is validated for both the screening and monitoring of cognitive impairment.^46,47^ Importantly, the MoCA demonstrates high sensitivity in detecting early signs of cognitive deficits, particularly when compared to the MMSE.^47-49^ Multiple aspects of cognition function are tested on the MoCA, including visuospatial/executive function, naming, delayed recall, attention, language, abstraction, and orientation, which add up to a total score of 30.

### Neurofilament Light Chain Assay

Blood samples were collected at baseline and 24-weeks and used to measure peripheral NfL protein levels. EDTA-treated plasma separator tubes were used for blood collection, and samples were then centrifuged at 1000 × g for 10-minute at room temperature (Model 614B, The Drucker 30 Company). Plasma supernatant was aliquoted and subsequently frozen at −80°C for analysis. NfL levels were quantified using a high-sensitivity sandwich ELISA method (AVIVA, OKCD01380).^50,51^ NfL levels were determined according to the manufacturers protocol using 96-well plates pre-coated with an antibody specific to NfL. Standards or samples diluted at 1:20 were added to the plate with a biotin-conjugated antibody. Avidin conjugated to horseradish peroxidase was added to each microplate well. 3,3',5,5'-Tetramethylbenzidine substrate was then added, producing an enzymatic reaction such that the wells containing NfL, biotin-conjugated antibody, and enzyme conjugated avidin changed colour. The colour change was measured using a standard microplate reader at a wavelength of 450 nm. Incubation steps and washes were done throughout the procedure as needed. Concentrations (pg/ml) were determined by comparing the optical density of the samples compared to the standard curve.

### Statistical Analyses

All statistical analyses were conducted using IBM SPSS Statistics (version 28.0) software. Data visualization was carried out using the Flexplot module on Jamovi (version 2.3.28). Continuous variables were summarized as means (SDs) and categorical variables were summarized as a count number (n) and percentage (%). Plasma NfL concentrations were assessed for normality using a Shapiro-Wilk test and visual inspection of the associated quantile-quantile plot. NfL levels were skewed and so a log_10_-transformation was applied to normalize the biomarker distribution prior to all analyses. Between group differences in demographic and clinical factors were assessed using independent sample t-tests for continuous variables and Chi-Square/Fisher’s exact tests for qualitative variables, respectively.

For the primary analysis, the baseline relationship between plasma NfL and MoCA scores in the vMCI group were assessed using a multivariable linear regression. Longitudinal changes in plasma NfL and MoCA scores over the 24-weeks of CR in this group were determined using a linear mixed model with time as a fixed factor and random subject-specific intercepts. In the secondary analyses, the association between both baseline and longitudinal plasma NfL and MoCA scores over time were assessed using similarly designed multivariable linear mixed models. The covariates for these within-subject multivariable analyses were chosen a priori, and included age, sex, and education. In addition, previous studies have found that greater CRF is associated with better cognitive outcomes in older adults.^52-54^ *Post-hoc* sensitivity analyses controlling for longitudinal VO_2peak_ as a covariate were therefore conducted to assess its potential influence on the associations between plasma NfL and MoCA scores over 24-weeks of CR. Finally, a one-way analysis of variance (ANOVA) was used to assess differences in plasma NfL levels between individuals with vMCI and age/sex matched CU controls. If between-group differences in demographic or clinical factors were found, they were controlled for as appropriate. Analyses were considered significant at a two-tailed α level of .05.

## Results

### Participant Characteristics

In total, 43 participants with vMCI were included in the baseline and longitudinal analyses. Baseline demographics and clinical characteristics are presented in Table 1. Additionally, data for between-group comparisons were available for a total of 39 age and sex-matched CU control participants. The vMCI group demonstrated significantly lower scores compared to controls on measures of global cognition (MoCA, MMSE) and individual tests within cognitive subdomains; however, no differences in any other clinical or demographic factor were found between groups.

**Table 1.** Demographic and Clinical Characteristics of Participants With Possible vMCI Included in the Study. Values Represent Mean ± SD or n, %.

	vMCI (n = 43)
Demographics	
Age	63.9 ± 6.7
Education (years)	16.0 ± 3.8
Sex (n, % male)	33, 76.7%
Clinical characteristics	
BMI (kg/m^2^)	29.5 ± 5.4
VO_2peak_ (mL⋅kg^−1^⋅min^−1^)	20.2 ± 5.8
NfL (pg/ml) ^2^	171.9 ± 56.1
Cognitive tests	
MMSE total	28.7 ± 1.2
MoCA total	24.9 ± 2.8
Vascular risk factors	
Hypertension	40, 93.0%
Obesity (BMI ≥30)	20, 46.5%
Current smoker	1, 2.3%
Diabetes mellitus	11, 25.6%
Coronary artery disease	43, 100.0%
Hyperlipidemia	43, 100.0%

BMI = Body Mass Index, MMSE = Mini-Mental State Examination, MoCA = Montreal Cognitive Assessment, VO_2peak_ (mL⋅kg^−1^⋅min^−1^) = peak oxygen uptake.

^a^NfL levels were log_10_ transformed prior to analysis.

### Plasma NfL Levels and MoCA Scores at Baseline

**Figure 1.**
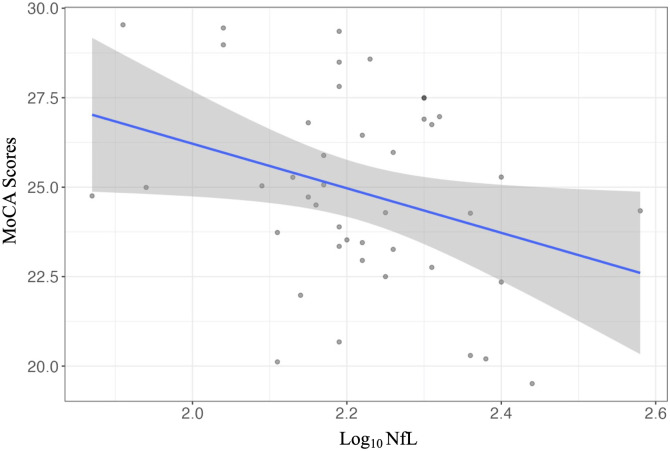
Association between NfL levels and MoCA scores in individuals with possible vMCI (n = 43) after adjusting for age, sex, and education as covariates.

An unadjusted bivariate regression analyses demonstrated that higher plasma NfL was associated with worse MoCA scores in those with vMCI (*r*(41) = −.344, *P* = .024). In multivariable linear regression analysis, plasma NfL was a significant predictor of MoCA scores (β = −.352 *P* = .029) (Figure 1). Specifically, higher NfL levels were associated with worse MoCA scores when controlling for age, sex, and education. Of note, no other predictors in the model were significantly associated with MoCA scores.

### NfL and MoCA Scores over Time

**Figure 2.**
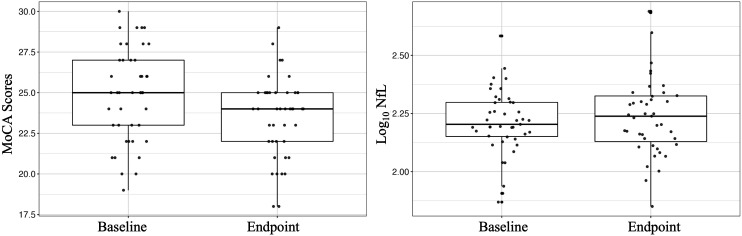
A boxplot showing participant MoCA scores (left) and Log_10_ NfL levels (right) at baseline and endpoint in n = 43 possible vMCI participants.

The distribution of MoCA and NfL scores at baseline and endpoint are presented visually in Figure 2. A total of 26 participants had longitudinally increased NfL levels (mean change (SD): .09 (.10) pg/ml) and 17 participants had either a decrease or no change (mean change (SD): −.08 (.05) pg/ml). However, there were no significant changes in log_10_ plasma NfL levels (baseline: 2.21 ± .14; endpoint 2.24 ± .17) over 24-weeks of CR (b[SE] = .03[.02], *P* = .134, df = 42). In regards to the MoCA, 11 participants had an increase in MoCA score (mean change (SD): 2.0 (1.1)), and 32 participants had a decrease or no change (mean change SD: −2.5 (2.1)). Furthermore, it was found that there was a significant decline in MoCA scores (baseline: 24.9 ± 2.8; endpoint: 23.5 ± 2.4) over time (*b*[SE] = −1.35[.42], *P* = .02, *df* = 42).

### Association Between Baseline NfL Levels and MoCA Scores Over Time

In a bivariate analysis, it was found that baseline NfL levels did not predict MoCA scores over time (*b*[SE] = −4.59[2.45], *P* = .068, *df* = 41). These results remained consistent after controlling for age, sex, and education (*b*[SE] = −4.80 [2.59], *P* = .072, *df* = 38). VO_2peak_ data at both baseline and endpoint were available for a total of 35 participants. Post-hoc sensitivity analyses controlling for change in VO_2peak_ (mL⋅kg^−1^⋅min^−1^) as an additional covariate demonstrated a significant association between higher plasma NfL level at baseline and a worsening in MoCA scores over time (*b*[SE] = −5.61 [2.73], *P* = .049, *df* = 31).

### Association Between Longitudinal NfL Levels and MoCA Scores Over Time

Increased plasma NfL levels were significantly associated with a decline in MoCA scores over time (*b*[SE] = −5.00 [1.96], *P* = .013, *df* = 68) in an unadjusted bivariate analysis. This relationship remained significant after controlling for age, sex, and education (Table 2). There was no change in the significance of the results after controlling for VO_2peak_, (*b*[SE] = −5.07 [2.14], *P* = .021, *df* = 58).

**Table 2.** Multivariable Linear Mixed Model of Change in NfL Levels Predicting MoCA Scores Over 24-Weeks of Cardiac Rehabilitation in n = 43 Possible vMCI Participants.

Fixed effect	Estimate (*b*)	SE	df^a^	*t*-value	*P*-value
NfL	−4.81	2.06	66	−2.33	.023
Age	.035	.054	40	.656	.516
Sex (male)	−.536	.800	38	−.673	.505
Education	.128	.094	39	1.37	.180

^a^Degrees of freedom estimated using Satterthwaite approximation.

### NfL Levels in vMCI and CU Controls

**Figure 3.**
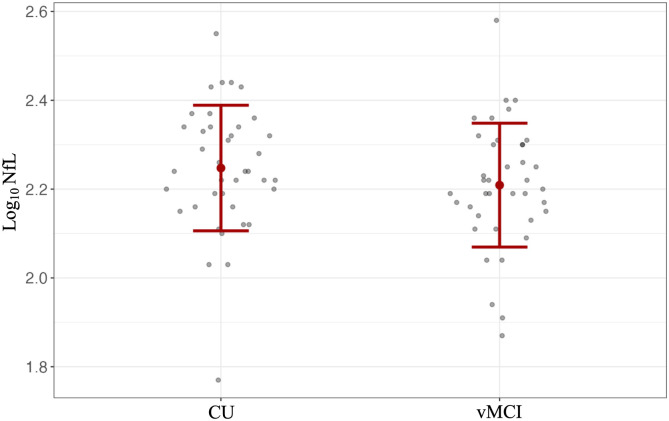
Log_10_ plasma NfL concentrations in the possible vMCI (n = 39) and age/sex-matched CU (n = 39) control group. Data are represented as mean ± SD.

The mean ± SD log_10_ plasma NfL levels were 2.25 ± .02 and 2.21 ± .02 in the vMCI and CU groups, respectively. No significant difference was found in plasma NfL levels between both groups at baseline (*F*(1, 76) = 1.37, *P* = .245) as shown in Figure 3. As previously mentioned, demographic and clinical characteristics between groups were comparable (Table 3), and so no adjustments for additional variables were made.

**Table 3.** Demographic and Clinical Characteristics of Possible vMCI and Age/Sex Matched CU Controls Included in the Cross-Sectional Study (n = 78). Values Represent Mean ± SD or n, %.

	vMCI (n = 39)	CU (n = 39)	*P*-value
Demographics			
Age	64.2 ± 6.9	64.0 ± 5.9	.899
Education	16.7 ± 2.5	15.6 ± 3.6	.130
Sex (n, % male)	33, 84.6%	33, 84.6%	1.00
Clinical characteristics			
BMI (kg/m^2^)	29.0 ± 5.4	29.5 ± 5.1	.639
VO_2peak_ (mL⋅kg^−1^⋅min^−1^)	21.2 ± 5.5	19.9 ± 5.5	.270
NfL (pg/ml) ^2^	170.1 ± 56.1	185.1 ± 56.7	.245
Cognitive tests			
MMSE	28.7 ± 1.2	29.3 ± 1.0	.025*
MoCA	24.9 ± 2.7	26.7 ± 2.1	.002*
CVLT-II z-score^a^	.36 ± .96	1.27 ± .77	<.001*
TMT-B z-score	−.60 ± .74	.38 ± .65	<.001*
COWAT z-score	−.40 ± .94	.45 ± .75	<.001*
Animal naming z-score	−.59 ± .76	.21 ± .64	<.001*
BVMT-R z-score	−.55 ± 1.07	.61 ± .74	<.001*
Vascular risk factors			
Hypertension	36, 92.3%	38, 97.4%	.615
Obesity (BMI ≥30)	18, 46.2%	14, 35.9%	.357
Current smoker	1, 2.6%	0, 0%	1.00
Coronary artery disease	39, 100%	39, 100%	1.00
Diabetes	10, 25.6%	5, 12.8%	.151
Hyperlipidemia	39, 100%	39, 100%	1.00

BMI = Body Mass Index, MMSE = Mini-Mental State Examination, MoCA = Montreal Cognitive Assessment, CVLT = California Verbal Learning Test-II, TMT-B = Trail-Making Test-B, COWAT = Controlled Oral Word Association Test, BVMT = Brief Visuospatial Memory Test - Revised.

*P-value <.05.

^a^n = 38 for the possible vMCI group.

## Discussion

The present study explored the utility of plasma NfL as a biomarker for participants at a high risk for vascular-related cognitive decline who were enrolled in a 24-week CR program. Specifically, we investigated whether NfL can be used as a predictive and monitoring biomarker of global cognition, as well as its diagnostic utility in a sample of individuals with vMCI.^55^ We employed both cross-sectional and longitudinal study designs to assess these overarching study goals. It was found that higher plasma NfL levels were significantly associated with worse MoCA scores (ie, global cognition) at baseline. Longitudinally, plasma NfL levels at baseline did not predict changes in global cognition over time; however, an increase in plasma NfL level was significantly associated with a decline in global cognition throughout the CR program. These findings remained significant after controlling for demographic factors (ie, age, sex, and education) in multivariable models. With regards to the diagnostic capacity of plasma NfL, no difference in NfL levels between both the vMCI and age/sex-matched CU control group was detected in this sample.

In previous literature, blood and CSF NfL were found to be associated with neuropathological changes (eg, cortical atrophy), as well as cerebrovascular pathology indicative of small vessel disease, such as cerebral microbleeds, white matter hyperintensities, and lacunar infarcts.^56-60^ In addition blood NfL levels have been cross-sectionally associated with cognition in clinical samples, including individuals with AD, VaD, and MCI.^61-65^ A recent meta-analysis reported that NfL and MMSE scores are negatively correlated in individuals with AD.^65^ The majority of studies to date have assessed the relationship between NfL and global cognition using tests such as the MMSE, Alzheimer’s Disease Assessment Scale–Cognitive Subscale (ADAS-Cog), or composite z-scores. In this study we used the MoCA as the primary measure of global cognition, due to evidence that it is sensitive to minor cognitive deficits, including executive deficits related to VCI.^46,47,66^ Furthermore, the MoCA is a short, and easy to administer examination, that is widely used across clinical settings. One previous study by Ma et al., 2020 found that higher serum NfL levels were associated with worse MoCA scores in individuals with VaD.^62^ Our finding that higher levels of plasma NfL are associated with worse MoCA scores at baseline in individuals with vMCI is therefore consistent with that study. Establishing a cross-sectional relationship between NfL and MoCA scores is thus an important step in evaluating its clinical utility as a biomarker of cognitive impairment in this participant population.

While cross-sectional studies provide important insight, longitudinal analyses are essential for a more in-depth understanding of the predictive capacity of NfL. Previous large-scale longitudinal analyses have found that higher blood NfL levels at baseline can predict worse cognitive outcomes over time in non-demented MCI or CU participants.^63,67-73^ For example, in the Mayo Clinic Study of Aging, it was reported that higher baseline plasma NfL was associated with a decline in composite global cognitive z-score, as well as domain-specific z-scores (eg, attention, memory, language) over time (median follow-up: 6.2 years) in a large participant cohort.^63^ These findings were replicated among participants from the Alzheimer’s Disease Neuroimaging Initiative (ADNI), in which they found that higher plasma NfL levels at baseline were associated with worse global cognition (measured by ADAS-Cog-13) over time (median follow-up: 3 years). In addition, 1 study in individuals with cerebral small vessel disease found that baseline NfL levels predicted cognitive decline and transition to dementia, with no change in NfL levels over time.^74^ Our results did not indicate that baseline plasma NfL levels could predict changes in MoCA scores over a 24-week time period in which all participants with vMCI were also concurrently undergoing CR. It is important to consider, however, that our sample size was much smaller than those previously mentioned studies with a shorter duration of follow up, making it difficult to detect small effect sizes. Furthermore, there might have been additional confounding factors that could influence cognitive outcomes in our sample. For instance, CR can improve CRF,^75-77^ albeit with variable effectiveness.^71,75,78^ While it is well established that an improvement in CRF is associated with better prognosis,^79-81^ several studies have also reported positive associations between CRF and cognition.^35,52-54,82^ Our *post-hoc* analysis controlling for VO_2peak_ as a confounding factor did reveal an association between higher baseline plasma NfL levels and worse global cognition over time, but it is of note that our sample size was not adequately powered for additional covariates.

This study also assessed the monitoring capacity of plasma NfL levels in those at risk for vascular-related cognitive decline by investigating the relationship between change in NfL levels and global cognition over 24-weeks of CR. The FDA-NIH BEST framework defines monitoring biomarkers as a “biomarker that can be measured repeatedly for assessing status of a disease or medical condition or for evidence of exposure to (or effect of) a medical product or an environmental agent”.^55^ Consistent with previous longitudinal studies in older adults with MCI and AD,^33,68,69^ we found that an increase in plasma NfL level was associated with a longitudinal decline in global cognition. Therefore, the present study supports the idea that plasma NfL can be used as an easily accessible blood-based biomarker to monitor changes in global cognition in individuals demonstrating mild cognitive deficits who are undergoing a CR program. This finding provides further insight into neuroaxonal injury as a potential neurobiological mechanism of cognitive decline in this vulnerable patient population.

Previous studies have reported elevated levels of plasma NfL among study participants with Alzheimer’s disease or MCI compared to healthy controls.^25,27-29,83,84^ Although the present study did not find that plasma NfL was elevated in the vMCI group compared to controls, it is important to consider that the control group in this study shared a similarly high vascular risk profile (eg, diabetes, hypertension, and CAD). It is possible that such underlying conditions could have led to cerebrovascular changes and neuronal injury that have not yet manifested in the form of cognitive impairment. Future analyses could be strengthened by including a cognitively normal control group free of vascular risk factors.

The present study has a number of limitations that could be addressed in future analyses. Firstly, a less strict criterion of at least 1 SD below the population norms on standardized assessments was used to classify cognitive impairment. This definition is consistent with the lower threshold suggested in both the DSM-5 and Gorelick criteria,^12,85^ but is less strict than the Petersen criteria, which require 1.5 SD below population norms for an MCI diagnosis.^9^ Thus, although the definition used here may increase sensitivity for detecting early cognitive deficits, it is possible that this may be at cost of specificity. In addition, the present study lacked neuroimaging confirmation of cerebrovascular disease. Participants were therefore classified as ‘possible’ vMCI rather than probable, as would be required in more recent consensus criteria.^45^

In the present study, participants with severely disrupted renal function were excluded, but continuous renal measures were not collected. Poor renal function is known to affect blood NfL levels,^86,87^ and so the inclusion of measures (eg, serum creatinine or estimated glomerular filtration rate) in future study protocols is warranted. Finally, this study was limited in terms of its relatively small sample size and the use of ELISA to quantify NfL levels as opposed to more novel ultrasensitive assays, such as single molecular array technology. Although all protein levels measured were within the limits of detection of the assay used, future studies should consider replicating the present findings in a larger sample using more recent ultrasensitive assays. Nonetheless, blood NfL allows for an easily accessible measure of neuroaxonal integrity, and so the inclusion of this biomarker in study protocols holds potential to increase understanding of neurobiological mechanisms in both observational and interventional studies. In addition to neuroaxonal injury, future protocols may also include other novel blood-based biomarkers of neurodegeneration (eg, glial fibrillary acidic protein and S100 calcium-binding protein B related to astroglial dysfunction). The addition of such markers may allow for a better characterization of neurodegeneration-related processes in prospective research studies.

In conclusion, higher plasma NfL levels were associated with worse global cognition at baseline in individuals with vMCI. Increased plasma NfL levels over time were also associated with a decline in global cognition over 24-weeks of CR in this at-risk population. However, we found limited evidence for the longitudinal predictive capacity of plasma NfL, and no evidence relating to the diagnostic capacity of NfL in our sample. Our study provides increased understanding of neuroaxonal injury as a potential mechanism of cognitive decline in individuals with vMCI. Future studies to replicate these findings should be conducted and may be strengthened by the use of more sensitive NfL assays in a larger sample with neuroimaging measures.

## Supplemental Material

Supplemental Material - Neurofilament Light Chain as a Biomarker of Global Cognition in Individuals With Possible Vascular Mild Cognitive Impairment Supplemental Material for Neurofilament Light Chain as a Biomarker of Global Cognition in Individuals With Possible Vascular Mild Cognitive Impairment by Amish Gaur, Damien Gallagher, Nathan Herrmann, Jinghan Jenny Chen, Susan Marzolini, Paul Oh, Yutaka Amemiya, Arun Seth, Alex Kiss, and Krista L. Lanctôt in Journal of Geriatric Psychiatry and Neurology
