# Supplementary material for: Neurofilament Light Chain as a Biomarker of Global Cognition in Individuals With Possible Vascular Mild Cognitive Impairment
Source: J Geriatr Psychiatry Neurol. 2024 May 17;38(1):62–72. doi: 10.1177/08919887241254469 (PMC11566096; doi:10.1177/08919887241254469)
Supplement: Supplemental Material - Neurofilament Light Chain as a Biomarker of Global Cognition in Individuals With Possible Vascular Mild Cognitive Impairment [file sj-pdf-1-jgp-10.1177_08919887241254469.pdf]

(Supplementary Information)

Neurofilament Light Chain as a Biomarker of Global Cognition in Individuals With Possible Vascular Mild Cognitive Impairment

Neuropsychological Evaluation

Verbal Memory

Verbal memory was assessed using the California Verbal Learning Test-II (CVLT-II) as it shows good re-test reliability[1,2] and high sensitivity/specificity for detecting amnestic MCI[3]. The following measures from the CVLT-II were used: verbal learning (5 learning trials in which a participant is asked to immediately recall a list of words) short delay free recall (word recall from a list that includes distractor words), and long delay free recall (recall of the original list of words after 20 minutes). Raw scores from the three tasks were converted to z-scores based on population norms and then averaged to create an overall z-score for the test.

Executive Function

Executive function was assessed using the trail-making test B (TMT-B) and controlled oral word association test (COWAT)[4–6]. Z-score were computed for each task based on population norms. The TMT-B test was chosen over the TMT-A as it is found to be both a sensitive and specific measure of executive function[7,8]. Additionally, the TMT-B task has shown good specificity in distinguishing MCI from controls[9,10]. In this task, participants are asked to draw a line from a number to a letter in ascending order (i.e., 1-A-2-B-3 and so on) as quickly as possible[7,11]. Higher elapsed times indicate worse performance on the test. In the COWAT, participants are required to name as many words as possible beginning with a certain letter (e.g., F, A, and S) within one minute[5,12]. The use of proper nouns or repeated stem words with different endings are not allowed. Although the COWAT is often considered to have good construct validity for executive functioning[4,6], it should be noted that it may also map well onto language domains[13]. Furthermore, studies have found that the COWAT can be used to distinguish between MCI and cognitively normal controls[14,15].

Language

Language function was evaluated using the animal naming test (semantic/categorical fluency)[5,6,13,16]. The animal naming test may also be considered to be a test of executive functioning[4], but regardless, it has shown to independently discriminate between MCI and normal cognition[15]. In this task, participants are asked to name as many animals as they can within 60 seconds[16]. A z-score for the test was computed based on population norms.

Visuospatial Function

Visuospatial abilities were assessed using the Brief Visuospatial Memory Test - Revised (BVMT-R), which involves an immediate visual learning and delayed recall component[17]. In the visual learning task, participants are given three 10-second learning trials in which they view a stimulus page and are asked to correctly draw as many figures as possible. In the delayed recall component, participants are asked to reproduce the original figures in the exact layout after 25-

minutes have elapsed. Z-scores for the two BVMT-R measures were computed based on population norms and then averaged.

References

1. Delis DC, Kramer JH, Kaplan E, Ober BA. California Verbal Learning Test--Second Edition. Published online November 14, 2016. doi:10.1037/t15072-000

2. Woods SP, Delis DC, Scott JC, Kramer JH, Holdnack JA. The California Verbal Learning Test – second edition: Test-retest reliability, practice effects, and reliable change indices for the standard and alternate forms. *Arch Clin Neuropsychol*. 2006;21(5):413-420. doi:10.1016/j.acn.2006.06.002

3. Rabin LA, Paré N, Saykin AJ, et al. Differential Memory Test Sensitivity for Diagnosing Amnestic Mild Cognitive Impairment and Predicting Conversion to Alzheimer's Disease. *Neuropsychol Dev Cogn B Aging Neuropsychol Cogn*. 2009;16(3):357-376. doi:10.1080/13825580902825220

4. Hachinski V, Iadecola C, Petersen RC, et al. National Institute of Neurological Disorders and Stroke-Canadian Stroke Network vascular cognitive impairment harmonization standards. *Stroke*. 2006;37(9):2220-2241. doi:10.1161/01.STR.0000237236.88823.47

5. Rosen WG. Verbal fluency in aging and dementia. *J Clin Neuropsychol*. 1980;2(2):135-146. doi:10.1080/01688638008403788

6. Freedman M, Leach L, Carmela Tartaglia M, et al. The Toronto Cognitive Assessment (TorCA): normative data and validation to detect amnestic mild cognitive impairment. *Alzheimers Res Ther*. 2018;10(1):65. doi:10.1186/s13195-018-0382-y

7. Arbuthnott K, Frank J. Trail making test, part B as a measure of executive control: validation using a set-switching paradigm. *J Clin Exp Neuropsychol*. 2000;22(4):518-528. doi:10.1076/1380-3395(200008)22:4;1-0;FT518

8. O'Sullivan M, Morris RG, Markus HS. Brief cognitive assessment for patients with cerebral small vessel disease. *J Neurol Neurosurg Psychiatry*. 2005;76(8):1140-1145. doi:10.1136/jnnp.2004.045963

9. Ashendorf L, Jefferson AL, O'Connor MK, Chaisson C, Green RC, Stern RA. Trail Making Test errors in normal aging, mild cognitive impairment, and dementia. *Arch Clin Neuropsychol Off J Natl Acad Neuropsychol*. 2008;23(2):129-137. doi:10.1016/j.acn.2007.11.005

10. Tamaru Y, Sumino H, Matsugi A. Usefulness of the Cognitive Composition Test as an Early Discriminator of Mild Cognitive Impairment. *J Clin Med*. 2023;12(3):1203. doi:10.3390/jcm12031203

11. Gaudino EA, Geisler MW, Squires NK. Construct validity in the Trail Making Test: what makes Part B harder? *J Clin Exp Neuropsychol*. 1995;17(4):529-535. doi:10.1080/01688639508405143

12. Faria C de A, Alves HVD, Charchat-Fichman H. The most frequently used tests for assessing executive functions in aging. *Dement Neuropsychol*. 2015;9(2):149-155. doi:10.1590/1980-57642015DN92000009

13. Whiteside DM, Kealey T, Semla M, et al. Verbal Fluency: Language or Executive Function Measure? *Appl Neuropsychol Adult*. 2016;23(1):29-34. doi:10.1080/23279095.2015.1004574

14. Bauer K, Malek-Ahmadi M. Meta-analysis of Controlled Oral Word Association Test (COWAT) FAS performance in amnestic mild cognitive impairment and cognitively unimpaired older adults. *Appl Neuropsychol Adult*. 2023;30(4):424-430. doi:10.1080/23279095.2021.1952590

15. McDonnell M, Dill L, Panos S, et al. Verbal fluency as a screening tool for mild cognitive impairment. *Int Psychogeriatr*. 2020;32(9):1055-1062. doi:10.1017/S1041610219000644

16. Goodglass H, Kaplan E. *The Assessment of Aphasia and Related Disorders*. 2nd ed.--. Lea & Febiger; 1983.

17. Benedict RHB, Groninger L, Schretlen D, Dobraski M, Shpritz B. Revision of the brief visuospatial memory test: Studies of normal performance, reliability, and, validity. *Psychol Assess*. 1996;8(2):145-153. doi:10.1037/1040-3590.8.2.145
